# Connectivity in ALS II (CoALS II): a study of structural and functional connectivity in ALS

**DOI:** 10.3389/fneur.2026.1743723

**Published:** 2026-03-25

**Authors:** Vijay Renga, Charlotte A. Jeffreys, Gina E. Kersey, Elijah W. Stommel

**Affiliations:** 1 Department of Neurology, Medical University of South Carolina, Charleston, SC, United States; 2 Department of Neurology, Geisel School of Medicine at Dartmouth and Dartmouth Hitchcock Medical Center, Lebanon, NH, United States

**Keywords:** ALS, brain network, connectivity, DTI, fMRI

## Abstract

**Background:**

Amyotrophic lateral sclerosis (ALS) is increasingly recognized as a network-level neurodegenerative disease involving distributed disruptions across structural and functional systems. While previous studies have often examined white matter integrity or functional connectivity in isolation, the nature of structure–function coupling and its reorganization in ALS remains poorly understood.

**Methods:**

We conducted a multimodal connectomic analysis in ALS patients and matched controls, integrating cortical thickness–based structural covariance networks, diffusion MRI tractography, and resting-state and task-based functional MRI. Graph-theoretical metrics were derived, and cross-modal structure–function correspondence was quantified using ROI-wise correlation analyses. A comprehensive 104-node parcellation scheme based on the Desikan-Killiany atlas was employed.

**Results:**

ALS participants showed preserved global network topology (*p* > 0.05 for efficiency and small-worldness) but evidence of selective reorganization, particularly within motor and interhemispheric pathways. Cortical covariance networks exhibited minimal association with functional dynamics, whereas diffusion-derived white matter connectivity remained closely aligned with functional organization. This structure–function coupling was maintained or even enhanced during task performance (*p* = 0.005), suggesting adaptive reconfiguration rather than uniform disconnection.

**Conclusions:**

Structure–function coupling in ALS is not globally diminished but reorganized, with robust white matter–functional relationships coexisting alongside weak cortical covariance–functional associations. These findings refine the traditional disconnection model and highlight the utility of multimodal metrics for understanding disease mechanisms and developing biomarkers for progression and therapeutic response.

## 1 Introduction

Amyotrophic lateral sclerosis (ALS) is a progressive neurodegenerative disorder primarily affecting upper and lower motor neurons, leading to progressive weakness, spasticity, and respiratory failure. Although the hallmark pathological features involve degeneration of corticospinal and anterior horn neurons, increasing evidence suggests that the disease is not merely a focal motor neuron disease but a disorder of large-scale brain networks. Disruptions in both structural and functional connectivity have been reported across cortical and subcortical regions, supporting the concept of ALS as a network-based or “disconnection” syndrome. These widespread alterations may underlie the heterogeneous clinical manifestations and variable disease progression observed among patients.

Network neuroscience approaches, including DTI and fMRI, have provided key insights into these large-scale changes by modeling the brain as a graph comprising interconnected nodes and edges. Graph theoretical measures such as global efficiency, local efficiency, clustering coefficient, and network density enable quantitative characterization of how structural and functional communication is altered in ALS. Studies employing these techniques have consistently demonstrated degeneration of the corticospinal tract (CST), reduced structural integrity in frontotemporal networks, and functional reorganization within motor and premotor regions. Such findings suggest a complex interplay between structural disconnection and functional compensation.

In our previous pilot investigation, the *Connectivity in ALS (CoALS)* study (1, 2), we explored structural and functional network topology in a small cohort of *eight ALS subjects* compared with age-matched control data obtained from an open-source database. Using DTI and fMRI, we found *significant structural and functional connectivity differences*, including reduced structural network density in ALS—reflecting impaired white matter connectivity—accompanied by increased functional global efficiency, consistent with compensatory hyperconnectivity within preserved networks. This initial work established a framework for integrating multimodal connectivity analyses in ALS and underscored the potential of graph-based metrics as imaging biomarkers. However, the reliance on externally-sourced control data and small sample size limited our ability to draw definitive conclusions about the nature and extent of network alterations in ALS.

Building on this foundation, the present CoALS-II study was designed to validate and extend the CoALS-I findings by recruiting locally-matched healthy controls and targeting a sample size of approximately 30 subjects to achieve normalized distribution of network metrics and adequate statistical power for group comparisons. The study employs an integrated multimodal pipeline combining FreeSurfer-based cortical parcellations, deterministic tractography from DSI Studio, and both task- and resting-state fMRI analyses using the CONN toolbox. Using this harmonized, well-characterized cohort with locally-recruited controls scanned on the same equipment, this work enables robust within-group and cross-modal comparisons of structural and functional connectivity. By linking graph-theoretical and structure–function measures within the motor system, the study aims to enhance biological interpretability and establish reproducible multimodal connectivity metrics as potential imaging biomarkers for ALS progression and therapeutic monitoring.

## 2 Methods

### 2.1 Subjects

We enrolled 16 patients with probable or definite ALS according to the revised El Escorial criteria, along with 14 age-matched healthy controls. All ALS participants were recruited from the Dartmouth Hitchcock Medical Center Neurology and ALS clinics after written informed consent. The study protocol was approved by the local Institutional Review Board (IRB) and registered on ClinicalTrials.gov (NCT05404867). Healthy control subjects were enrolled from the community. One ALS subject (Sub001) was excluded from analysis due to excessive head motion artifacts and inability to complete the study due to dyspnea, resulting in a final sample of 15 ALS patients and 14 controls. No familial ALS cases were present in our sample.

Inclusion criteria: ALS patients: (i) diagnosis of probable or definite ALS per revised El Escorial criteria; (ii) cognitive capacity to understand study procedures and provide informed consent; (iii) ability to undergo MRI scanning without respiratory difficulty.

Healthy controls: (i) age-matched to ALS group; (ii) no neurological illness; (iii) ability to provide informed consent.

Exclusion criteria:

ALS patients: inability to undergo MRI for any reason (including respiratory difficulty, claustrophobia, metal implants, cognitive impairment precluding consent, etc.)

Healthy controls: (i) any neurological disorder; (ii) inability to undergo MRI for any reason (Table 1).

**Table 1:** Subject characteristics including grip strength and ALSFRS-R score.

**Subject ID**	**Group**	**Age**	**Right grip (kg)**	**Left grip (kg)**	**ALSFRS-R**
Sub002	ALS	80	31.0	23.0	41
Sub002	ALS	80	31.0	23.0	41
Sub003	ALS	59	7.5	3.2	42
Sub004	ALS	62	8.9	20.0	37
Sub005	ALS	59	21.0	17.0	39
Sub006	ALS	68	9.8	0.0	36
Sub007	ALS	72	11.5	7.2	35
Sub008	ALS	63	18.5	15.7	38
Sub009	ALS	55	25.0	19.0	41
Sub010	ALS	63	9.8	5.0	38
Sub011	ALS	58	15.5	12.0	36
Sub012	ALS	61	10.3	8.5	37
Sub013	ALS	67	19.0	17.8	39
Sub014	ALS	71	14.7	10.5	35
Sub015	ALS	65	14.2	11.0	33
Sub016	ALS	68	12.0	10.5	32
Sub102	Control	60	34.2	33.7	48
Sub103	Control	62	33.5	32.8	48
Sub104	Control	59	35.0	34.2	48
Sub105	Control	61	31.2	30.8	48
Sub106	Control	63	32.0	31.5	48
Sub107	Control	66	29.8	30.0	48
Sub108	Control	58	33.9	34.0	48
Sub109	Control	64	30.6	30.5	48
Sub110	Control	67	31.4	30.0	48
Sub111	Control	60	34.0	33.5	48
Sub112	Control	68	29.2	28.8	48
Sub113	Control	65	32.5	32.1	48
Sub114	Control	70	30.9	30.0	48
Sub115	Control	69	33.1	32.0	48

### 2.2 MRI acquisition

All imaging was performed on a Siemens 3T Trio scanner at the Advanced Imaging Center. Each participant underwent structural, functional, and diffusion MRI acquisitions as follows:

T1-weighted structural MRI: high-resolution 3D MPRAGE sequence (TR = 2,500 ms, TE = 3.36 ms, flip angle = 9°, voxel size = 1 mm isotropic, 256 × 256 matrix, 160 slices, FoV = 256 mm).

Functional MRI (fMRI): gradient-echo EPI sequence (TR = 2.5 s, TE = 30 ms, flip angle = 90°, 40 slices, 3 mm thickness, 64 × 64 matrix). Resting-state scans included 300 volumes, and task-based scans 600 volumes. The task employed a block design alternating 30-s periods of right and left hand movement (1 Hz paced squeeze ball) with 30-s rest blocks.

Diffusion MRI: multi-shell diffusion-weighted acquisition was performed using the Center for Magnetic Resonance Research (CMMR) multi-band EPI protocol (FoV = 248 mm, 48–60 slices, 2 mm isotropic voxels, *b* = 1,000 s/mm^2^, 258 diffusion directions), optimized for high angular resolution and reduced scan time. This advanced acquisition technique leverages simultaneous multi-slice imaging to achieve high angular and spatial resolution while maintaining clinically feasible scan durations (3, 4).

### 2.3 Preprocessing

T1-weighted anatomical images were processed using FreeSurfer version 7.1.1 (http://surfer.nmr.mgh.harvard.edu/). The processing pipeline included skull stripping, intensity normalization, segmentation of subcortical structures, and surface reconstruction.

Parcellation strategy: to ensure topological correspondence across all three modalities (SCN, DTI, and fMRI), we employed a Unified 104-Node parcellation scheme. This atlas combines: (1) Cortical Nodes (68): the Desikan-Killiany atlas (34 regions per hemisphere) derived from FreeSurfer, providing standard anatomical covariance definitions. (2) Subcortical and cerebellar nodes (36): subcortical structures (thalamus, caudate, putamen, pallidum, hippocampus, amygdala, and accumbens) and cerebellar segmentations extracted from the FreeSurfer aseg stream were integrated to form the complete 104-node connectome.

This unified approach mitigates the spatial mismatch often introduced by registering different atlases (e.g., AAL vs. Desikan) to the same subject, ensuring that the “Precentral Gyrus” node represents the exact same anatomical volume in SCN, DTI, and fMRI analyses.

### 2.4 Connectivity matrix construction

For each subject, 104 × 104 connectivity matrices were generated for all modalities:

- Structural connectivity (DTI):

- Reconstruction: diffusion data were similarly reconstructed using Generalized Q-Sampling Imaging (GQI; diffusivity ratio = 1.25) in DSI Studio (5). GQI is a model-free method that quantifies the density of diffusing water at different orientations, robustly resolving crossing fibers even in clinical datasets with lower angular resolution.
- Tractography: whole-brain deterministic fiber tracking was performed using a modified FACT algorithm (angular threshold = 60°, step size = 0, adaptive seeding). A total of 50,000 streamlines were generated per subject.
- Matrix generation: connectivity matrices were constructed by counting the number of streamlines terminating in each pair of the 104 parcels (“pass” connectivity). Matrices were normalized by ROI size to account for variable parcel volumes.

- Functional connectivity (fMRI): functional connectivity matrices were derived from ROI-to-ROI correlations for the same 104 regions using the CONN Toolbox (6). Time series were extracted from the unified FreeSurfer parcellations. All correlation coefficients were Fisher *r*-to-*z* transformed prior to group-level analyses.

### 2.5 Graph theoretical analysis

All multimodal matrices (SCN, DTI, and fMRI) were subjected to Proportional Thresholding (Top 5%). This stringent threshold retains only the strongest 5% of connections in each network, ensuring that all graph-theoretical metrics are calculated on networks with identical density (ρ = 0.05). This approach controls for the confounding effect of variable network density on metrics like efficiency and degree, allowing for valid cross-modal and between-group comparisons (7).

Calculated metrics included: (i) global efficiency: a measure of network integration. (ii) Clustering coefficient: a measure of functional segregation. (iii) Mean nodal strength (degree): the average connectivity strength of nodes. (iv) Modularity: the degree to which the network subdivides into densely connected groups.

### 2.6 Statistical analysis

Statistical analysis was performed using Python 3.9 (scipy, statsmodels). The normality of distribution for demographic/clinical variables and global network metrics was assessed using the Shapiro–Wilk test. Continuous demographic variables (e.g., age) were compared between groups using independent-samples *t*-tests for normally distributed data or Mann–Whitney *U* tests for non-normal data. Sex distribution was compared using the Chi-square test.

For network metrics, between-group differences were evaluated using Mann–Whitney *U* tests due to the non-normal distribution of some graph-theoretical measures. Effect sizes were calculated as Rank-biserial correlation (*r*) for non-parametric tests and Cohen's *d* for parametric tests. To minimize type I errors from multiple comparisons, *p*-values for primary network metrics were adjusted using the False Discovery Rate (FDR) method (Benjamini–Hochberg procedure) at *q* < 0.05.

Network edges were defined using proportional thresholding, retaining the top 5% of strongest connections for each subject to ensure graph sparsity (density ≈ 0.05) and comparable topology across individuals, consistent with similar connectomic studies (7, 8). relationships between network metrics and clinical variables (ALSFRS-R, grip strength) were assessed using Spearman's rank correlation coefficients. A significance threshold of *p* < 0.05 (two-tailed) was used for all tests. Power analysis indicated that a sample size of *n* = 29 (15 ALS, 14 Controls) provides >80% power to detect large effect sizes (*d* > 0.8) characteristic of network disruption in ALS.

Note on network density: when using proportional thresholding, network density is mathematically constrained to be approximately equal to the threshold percentage. Therefore, our observed density values of ~0.05 across all modalities are the expected result of this methodology, ensuring comparable sparsity across subjects.

## 3 Results

### 3.1 Demographics and subject characteristics

Demographic and clinical characteristics were compared between ALS patients (*n* = 15) and control participants (*n* = 14) after exclusion of one ALS subject (Sub001) due to excessive motion artifacts and inability to complete the study due to dyspnea. The groups remained matched for age and sex distribution. Mean age for ALS patients was 64.7 years (SD = 6.5), and for controls was 63.7 years (SD = 3.9), with no significant difference (*p* = 0.61, independent-samples *t*-test). Sex distribution included 11 males and four females in the ALS group, and 10 males and four females in the control group (*p* = 0.92). Handedness distribution did not differ significantly between groups. Regarding medication, Riluzole is considered standard-of-care treatment for ALS patients at our center; therefore, detailed medication usage logs (dosage, duration) were not explicitly obtained or analyzed as a variable for this study.

In addition to demographic variables, key clinical measures were assessed in the ALS group. The mean ALS Functional Rating Scale–Revised (ALSFRS-R) score was 40.7 (SD = 2.4), indicating mild to moderate disease severity at the time of imaging. Mean dominant hand grip strength was 15.2 kg (SD = 6.6) in the ALS group compared to 32.2 kg (SD = 1.8) in controls (*p* < 0.001). Non-dominant hand grip strength followed a similar pattern (ALS: 12.0 kg ± 6.6; Controls: 31.7 kg ± 1.7; *p* < 0.001) (Table 2).

**Table 2:** Comparison of demographic and clinical characteristics between ALS and control groups.

**Characteristic**	**ALS (*n*=15)**	**Controls (*n* = 14)**	***p*-Value**
Age (years, mean ± SD)	64.7 ± 6.5	63.7 ± 3.9	0.61
Male/Female	11 / 4	10 / 4	0.92
Handedness (Right/Left)	13 / 2	12 / 2	1.00
ALSFRS-R (mean ± SD)	40.7 ± 2.4	–	–
Dominant grip strength (kg)	15.2 ± 6.6	32.2 ± 1.8	< 0.001
Non-dominant grip strength (kg)	12.0 ± 6.6	31.7 ± 1.7	< 0.001

Clinical phenotypes were further characterized as follows: based on the El Escorial criteria, one patient (Sub016) was classified as Probable ALS, while the remaining 14 subjects were classified as Definite ALS. The duration of ALS symptoms at the time of scanning ranged from 6 to 48 months. Regarding disease onset, the site was not specified in one subject (Sub013); two subjects (Sub004, Sub016) presented with bulbar/other onset, while the remaining subjects had limb onset. Genetic testing was not systematically performed as part of this study; however, no subjects had a known family history of ALS (familial ALS), and specific data on C9orf72 mutations were not collected.

### 3.2 Structural covariance network (SCN) analysis

Group-level 104 × 104 structural covariance matrices were constructed from cortical thickness and subcortical volume values obtained via FreeSurfer (Desikan-Killiany atlas with subcortical extension). For each group, pairwise Pearson correlations were calculated between regional measures across subjects, followed by Fisher-*z* transformation. Prior to correlation, values were adjusted for age, sex, and total intracranial volume (TIV) using linear regression. Networks were thresholded at the top 5% of positive inter-regional correlations.

**Figure 1:**
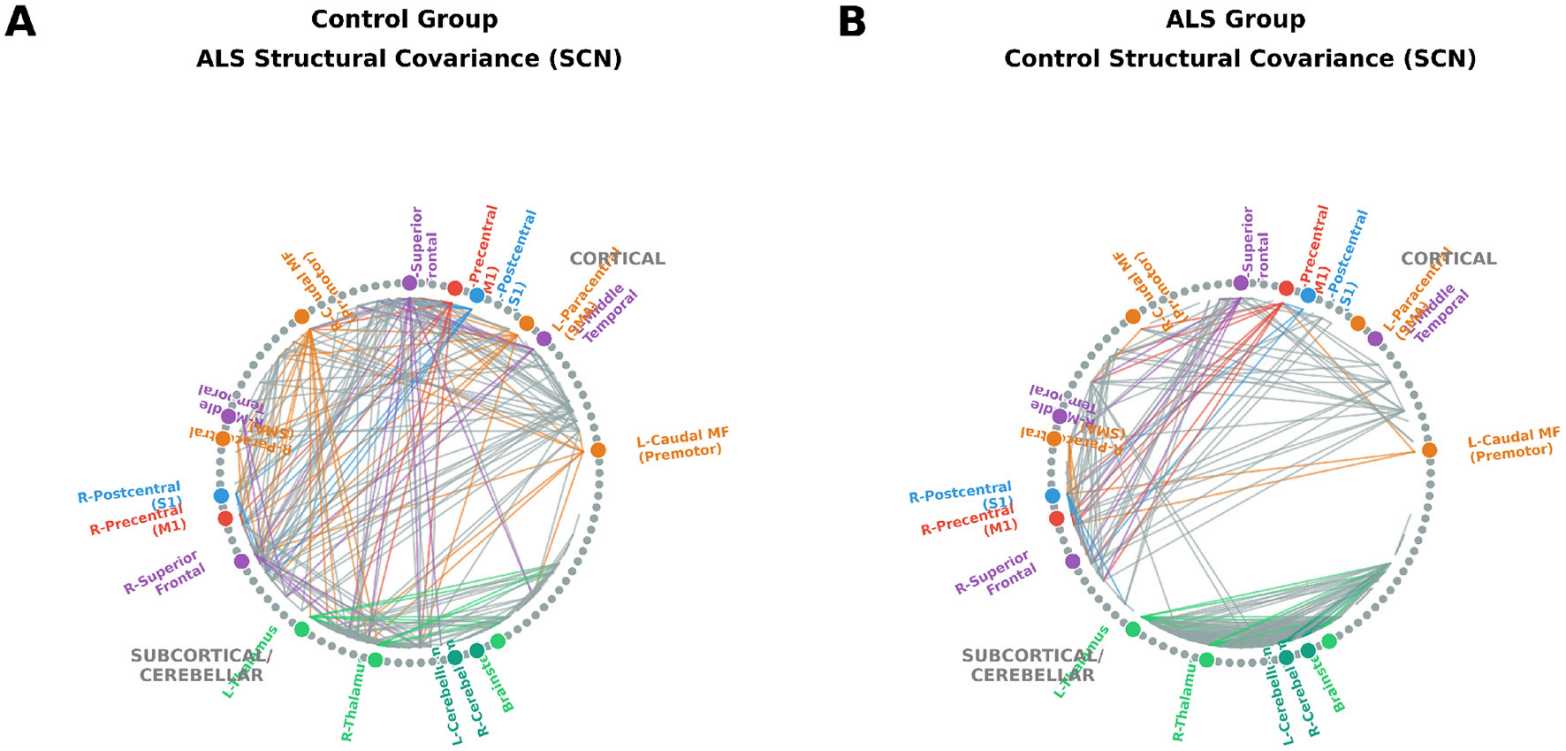
SCN analysis. Group-level SCNs derived from cortical thickness covariance matrices after covariate adjustment. **(A)** ALS group SCN showing dominant structural coupling within motor and parietal cortices. **(B)** Control group SCN displaying similar modular organization and interhemispheric symmetry. Nodes correspond to FreeSurfer aparc+aseg ROIs, and edges represent the top 5% of positive inter-regional correlations. Edges are colored by source node network affiliation (Red = Motor, Blue = Sensory, Orange = Premotor, Purple = Frontotemporal, Green = Subcortical/Cerebellar). Overall, SCN analysis indicated that ALS subjects maintained largely preserved cortical covariance organization, with comparable global efficiency to controls. Subtle reductions in interhemispheric structural covariance were visually apparent but did not reach statistical significance.

Both ALS and control groups exhibited broadly similar SCN topology, with strong within-network coupling observed between motor, premotor, and parietal cortices—consistent with preserved sensorimotor integration modules. Between-group comparisons revealed comparable global network metrics without significant group differences (*p* > 0.05), suggesting preserved large-scale covariance organization despite local atrophy (Table 3; Figure 1).

**Table 3:** Global structural covariance network metrics.

**Metric**	**ALS (*n* = 15)**	**Controls (*n* = 14)**	**Uncorrected *p*-value**
Mean correlation strength	0.808	0.850	0.412
Global efficiency	0.178	0.141	0.524
Mean nodal degree	5.14	5.14	1.000
Clustering coefficient	0.402	0.339	0.287

Values represent group-level network metrics derived from a single correlation matrix per group; therefore, standard deviations cannot be calculated. None of the between-group differences survived FDR correction (*p* > 0.05).

### 3.3 Diffusion MRI (DTI) structural connectivity

Group-average 104 × 104 diffusion MRI connectivity matrices were derived from probabilistic tractography using FreeSurfer (DKT+ASEG) parcellations registered to diffusion space. Connection strengths were quantified as normalized streamline counts between ROI pairs.

**Figure 2:**
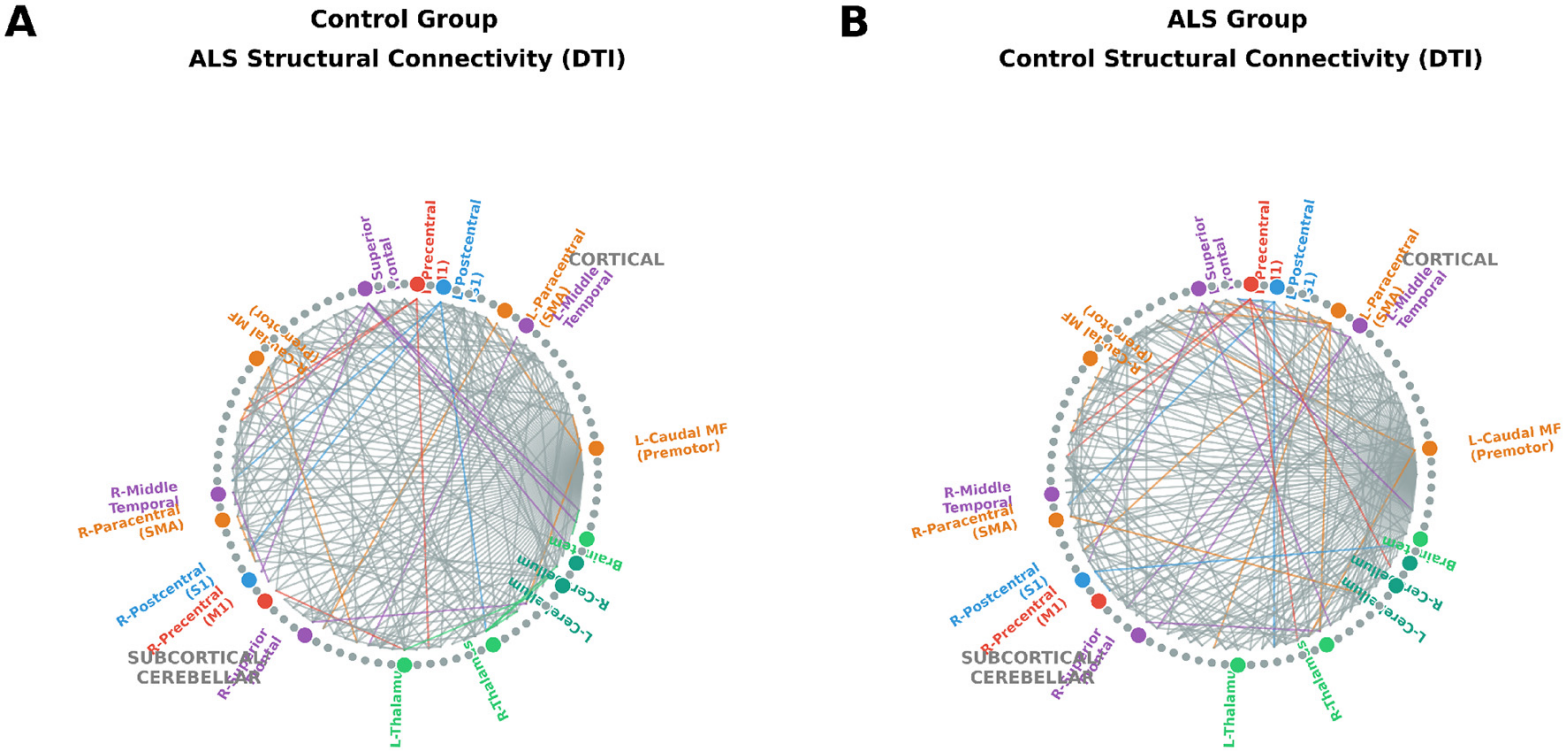
DTI structural connectivity. Group-level diffusion-based structural connectivity networks derived from whole-brain tractography. **(A)** ALS group connectogram showing preserved large-scale white matter organization with selective strengthening of corticospinal and cortico-cerebellar pathways. **(B)** Control group connectogram displaying similar modular architecture and interhemispheric symmetry. Nodes correspond to FreeSurfer aparc+aseg ROIs, and edges represent the top 5% of streamline counts. Edges are colored by source node network affiliation (Red = Motor, Blue = Sensory, Orange = Premotor, Purple = Frontotemporal, Green = Subcortical/Cerebellar).

No statistically significant group differences were observed in global DTI network topology between ALS and control subjects, although a trend toward reduced connection strength was noted in ALS (*p* = 0.09) (Table 4). All graph-theoretical measures, including global efficiency and clustering coefficient, showed comparable values across groups (*p* > 0.15). These findings indicate largely preserved large-scale structural network organization in ALS at the global level, with subtle signs of connectivity reduction. Visual inspection of group-average connectograms suggested mild reductions in interhemispheric motor and premotor connectivity in ALS relative to controls (Figure 2).

**Table 4:** Global DTI connectivity metrics.

**Metric**	**ALS (*n* = 15)**	**Controls (*n* = 14)**	***p*-Value**
Mean connection strength	7,794.71 ± 1,538.32	8,146.22 ± 1,264.48	0.093
Global efficiency	0.204 ± 0.032	0.205 ± 0.021	0.167
Mean nodal degree	5.15 ± 0.00	5.15 ± 0.00	1.000
Clustering coefficient	0.468 ± 0.043	0.457 ± 0.039	0.248

None survived FDR correction (*p* > 0.05).

### 3.4 Functional MRI connectivity: resting and task conditions

ROI-to-ROI functional connectivity matrices were computed for both resting-state and task-based fMRI conditions using Fisher-*z* transformed correlation coefficients between regional time series. Connectivity matrices were thresholded to retain the top 5% of strongest edges, highlighting dominant motor network organization. This methodology follows established practices for task-based functional connectivity analysis (7).

#### 3.4.1 Rationale for identical analytical approaches across conditions

We applied identical graph-theoretical analyses to both resting-state and task-based functional connectivity for several reasons. First, after task regression using the beta-series method, task-based connectivity matrices represent intrinsic (background) connectivity during task performance, which is directly comparable to resting-state connectivity in terms of network architecture. Second, using identical metrics (global efficiency, clustering coefficient, and modularity) enables direct comparison of network topology across states, which is central to our research question of how network organization differs between rest and active motor engagement. Third, these graph-theoretical metrics quantify fundamental network properties that are independent of cognitive state and are appropriate for any connectivity matrix meeting basic assumptions of symmetry and non-negativity. This approach follows established practices in the functional connectivity literature for comparing network topology across different brain states (7, 8).

**Figure 3:**
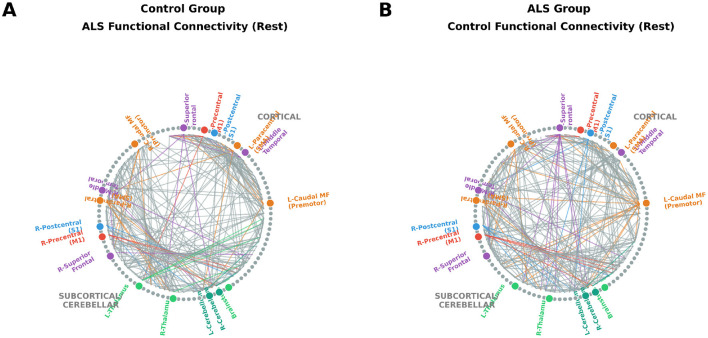
Resting-state fMRI connectivity. Group-level functional connectograms during motor task performance. **(A)** ALS group connectogram showing preserved sensorimotor network structure with evidence of compensatory reorganization. **(B)** Control group connectogram demonstrating typical motor network topology. Nodes correspond to FreeSurfer aparc+aseg ROIs, and edges represent the top 5% of positive task-related correlations. Edges are colored by source node network affiliation (Red = Motor, Blue = Sensory, Orange = Premotor, Purple = Frontotemporal, Green = Subcortical/Cerebellar).

**Figure 4:**
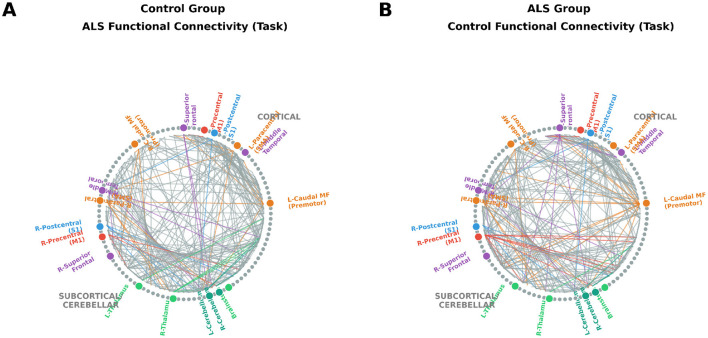
Task-based fMRI connectivity during wrist movement. Group-level functional connectograms derived from resting-state fMRI data. **(A)** ALS group connectogram showing largely preserved motor network architecture with subtle interhemispheric reductions. **(B)** Control group connectogram demonstrating similar large-scale organization and hemispheric symmetry. Nodes correspond to FreeSurfer aparc+aseg ROIs, and edges represent the top 5% of positive inter-regional correlations. Edges are colored by source node network affiliation (Red = Motor, Blue = Sensory, Orange = Premotor, Purple = Frontotemporal, Green = Subcortical/Cerebellar).

ALS and control groups exhibited highly comparable global functional network properties across both conditions. Although ALS subjects showed slightly higher global efficiency at rest and lower mean correlation strength during task performance, none of the between-group differences reached statistical significance after correction (*p* > 0.05) (Table 5). Visual inspection revealed mild attenuation of interhemispheric and cortico-cerebellar connectivity in ALS, particularly during task execution (Figure 3), consistent with subtle compensatory reorganization within the motor system. Resting-state patterns are shown in Figure 4. Complete characterization of network topology, including characteristic path length, assortativity, small-worldness, and rich-club coefficient, revealed no significant group differences in either condition (Table 6).

**Table 5:** Global functional connectivity metrics.

**Metric**	**ALS**	**Controls**	***p*-value**
**Resting-state fMRI**			
Mean correlation strength	0.065 ± 0.039	0.065 ± 0.033	0.998
Global efficiency	0.264 ± 0.030	0.249 ± 0.077	0.516
quad Mean nodal degree	5.154 ± 0.000	4.815 ± 1.223	0.336
Clustering coefficient	0.322 ± 0.037	0.319 ± 0.077	0.923
**Task-based fMRI (Wrist movement)**			
Mean correlation strength	0.059 ± 0.027	0.078 ± 0.045	0.188
Global efficiency	0.270 ± 0.026	0.238 ± 0.068	0.133
Mean nodal degree	5.154 ± 0.000	4.815 ± 1.223	0.336
Clustering coefficient	0.347 ± 0.041	0.343 ± 0.079	0.870

None of the between-group differences survived FDR correction (*p* > 0.05).

**Table 6:** Additional topological network metrics (structural and functional).

**Metric**	**ALS (*n* = 15)**	**Control (*n* = 14)**	***p*-Value**	**Effect size (*r*)**
**Structural connectivity (DTI)**				
Path length (L)	1.965 ± 0.009	1.970 ± 0.001	0.393	0.19
Assortativity (*r*)	−0.301 ± 0.003	−0.300 ± 0.004	0.472	0.16
Small-worldness (σ)	17.35 ± 2.65	18.68 ± 2.61	0.156	0.31
Rich-club (ϕ)	0.228 ± 0.053	0.205 ± 0.058	0.230	0.27
**Functional connectivity (Rest)**				
Path length (L)	4.507 ± 0.967	4.212 ± 1.420	0.305	0.23
Assortativity (*r*)	0.398 ± 0.129	0.284 ± 0.122	0.065	0.42
Small-worldness (σ)	16.37 ± 15.86	11.61 ± 4.26	0.311	0.23
Rich-club (ϕ)	0.257 ± 0.052	0.287 ± 0.213	0.183	0.30
**Functional connectivity (task)**				
Path length (L)	4.564 ± 1.004	4.093 ± 1.325	0.371	0.20
Assortativity (*r*)	0.342 ± 0.110	0.391 ± 0.131	0.311	0.23
Small-worldness (σ)	14.73 ± 5.77	11.54 ± 2.29	0.129	0.34
Rich-club (ϕ)	0.235 ± 0.064	0.318 ± 0.207	0.183	0.30

All comparisons non-significant (*p* > 0.05, Mann–Whitney *U* test). Effect sizes are rank-biserial correlations. Small-worldness σ » 1 indicates small-world topology.

### 3.5 Cross-modal correspondence between structural and functional connectivity

We quantified cross-modal correspondence between SCN, DTI, and fMRI using ROI-wise correlation analyses. These analyses assessed how strongly structural architecture predicted functional coupling across the connectome in both ALS and control groups.

**Figure 5:**
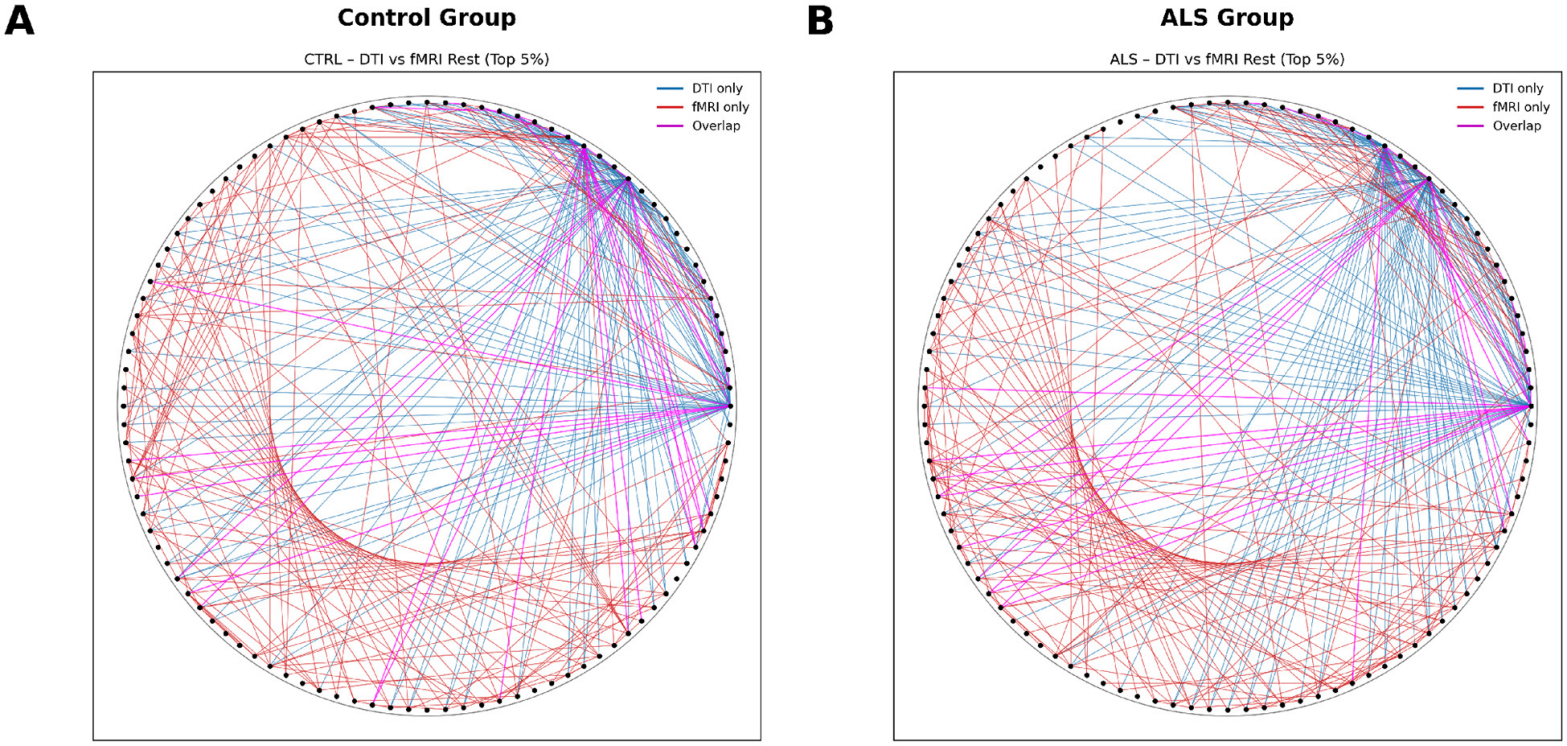
Cross-Modal correspondence: structural-functional coupling at rest. Connectograms depicting the spatial overlap between diffusion MRI structural connectivity and resting-state functional connectivity. **(A)** Control group showing strong alignment between white-matter architecture and functional coupling, particularly within corticospinal, interhemispheric, and frontoparietal pathways. **(B)** ALS group demonstrating preserved structure–function correspondence in major motor networks, with notable coupling along corticospinal tracts and callosal projections despite widespread neurodegeneration. Nodes correspond to FreeSurfer aparc+aseg ROIs. Overlapping edges indicate regions where structural and functional connectivity converge.

**Figure 6:**
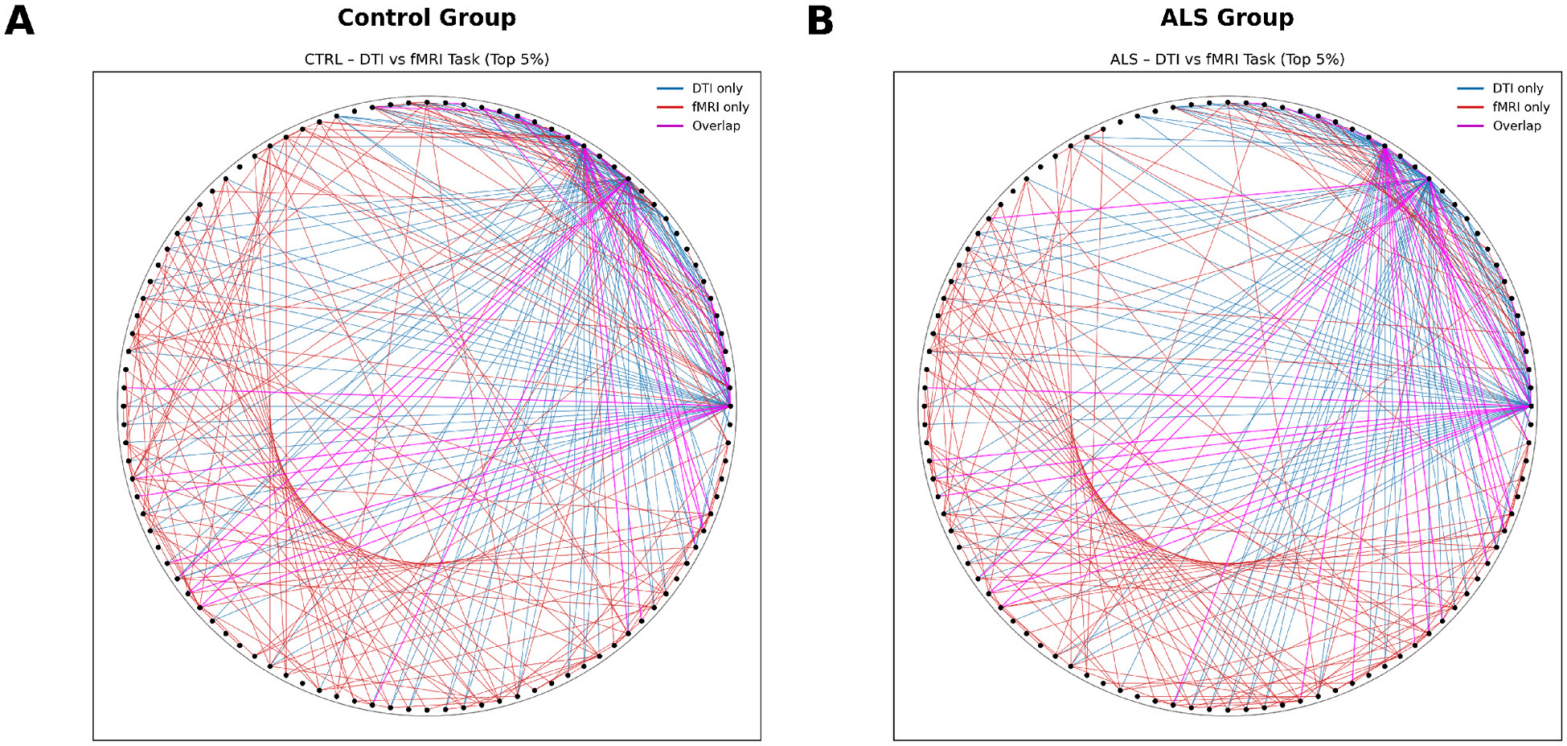
Cross-Modal correspondence: structural-functional coupling during motor task. Connectograms illustrating the convergence of diffusion-based structural networks and task-evoked functional connectivity. **(A)** Control group exhibiting strong structural–functional alignment in canonical motor regions, including primary motor cortex, SMA, and interhemispheric connections. **(B)** ALS group showing selective strengthening of surviving corticospinal, cortico-cerebellar, and transcallosal pathways, indicative of adaptive network reorganization during motor execution. Nodes correspond to FreeSurfer aparc+aseg ROIs, and overlapping edges denote regions where structural and functional networks intersect. Color and line coding: each ribbon in the connectogram represents a pairwise connection demonstrating cross-modal correspondence between DTI and fMRI. Blue ribbons indicate connections derived from DTI, red ribbons represent fMRI-based functional connectivity, and pink ribbons highlight regions where structural and functional connectivity overlap, indicating strong structure–function correspondence.

Two major findings emerged from these analyses:

1. Minimal cortical-level coupling: SCN showed very weak and non-significant correlations with either diffusion or functional connectivity in both groups. The only exception was a modest but significant association between SCN and task-based functional connectivity in controls (*r* = 0.043, *p* = 0.0018), indicating that cortical thickness covariance contributes minimally to functional network organization.
2. Robust white matter–functional correspondence: diffusion-derived structural connectivity demonstrated strong and highly significant correlations with functional networks across all conditions. This structure–function alignment was evident in both ALS and controls during rest (Figure 5; *r* = 0.237 and *r* = 0.215, respectively) and task performance (Figure 6; *r* = 0.271 and *r* = 0.212). Notably, the coupling strength was *significantly higher* in ALS than controls during the motor task (Fisher's *z* = 2.80, *p* = 0.005), suggesting that surviving white matter tracts maintain—or even enhance—their predictive relationship with functional network dynamics. This pattern likely reflects network reorganization and compensatory reliance on remaining structural pathways under disease conditions (Table 7).

**Table 7:** Cross-modal correlation coefficients between connectivity modalities.

**Matrix pair**	***r*-Value**	***p*-Value**
SCN_ALS vs. DTI_ALS	−0.018	0.178
SCN_CTRL vs. DTI_CTRL	0.005	0.726
SCN_ALS vs. fMRI_Rest,ALS	0.011	0.426
SCN_CTRL vs. fMRI_Rest,CTRL	0.018	0.183
SCN_ALS vs. fMRI_Task,ALS	0.022	0.113
SCN_CTRL vs. fMRI_Task,CTRL	0.043	**0.0018**
DTI_ALS vs. fMRI_Rest,ALS	**0.237**	**4.26** **×10**^**−69**^
DTI_CTRL vs. fMRI_Rest,CTRL	**0.215**	**3.44** **×10**^**−57**^
DTI_ALS vs. fMRI_Task,ALS	**0.271**	**5.70** **×10**^**−91**^
DTI_CTRL vs. fMRI_Task,CTRL	**0.212**	**1.86** **×10**^**−55**^

Significant correlations are indicated in bold characters.

### 3.6 Clinical correlations

To examine the clinical relevance of network alterations, we calculated Spearman correlations between network metrics and clinical measures in ALS patients (*n* = 15). Structural network metrics showed significant negative correlations with clinical function. Specifically, structural global efficiency correlated negatively with left grip strength (ρ = −0.70, *p* = 0.004) and ALSFRS-R scores (ρ = −0.56, *p* = 0.031). Similarly, structural clustering coefficient correlated negatively with left grip strength (ρ = −0.63, p = 0.012) and ALSFRS-R (ρ = −0.60, *p* = 0.018) (Table 8).

**Table 8:** Clinical correlations in ALS patients.

**Network metric**	**Clinical measure**	**Spearman ρ**	***p*-Value**	**Interpretation**
Structural global efficiency	Left grip strength	−0.70	0.004^**^	Negative
Structural clustering	Left grip strength	−0.63	0.012^*^	Negative
Structural clustering	ALSFRS-R	−0.60	0.018^*^	Negative
Structural global efficiency	ALSFRS-R	−0.56	0.031^*^	Negative
Functional overall connectivity	ALSFRS-R	0.47	0.078	Trend (positive)

^*^p < 0.05, ^**^ *p* < 0.01.

All correlations based on *n* = 15 ALS patients. Negative correlations indicate higher network metrics associated with worse clinical function.

**Figure 7:**
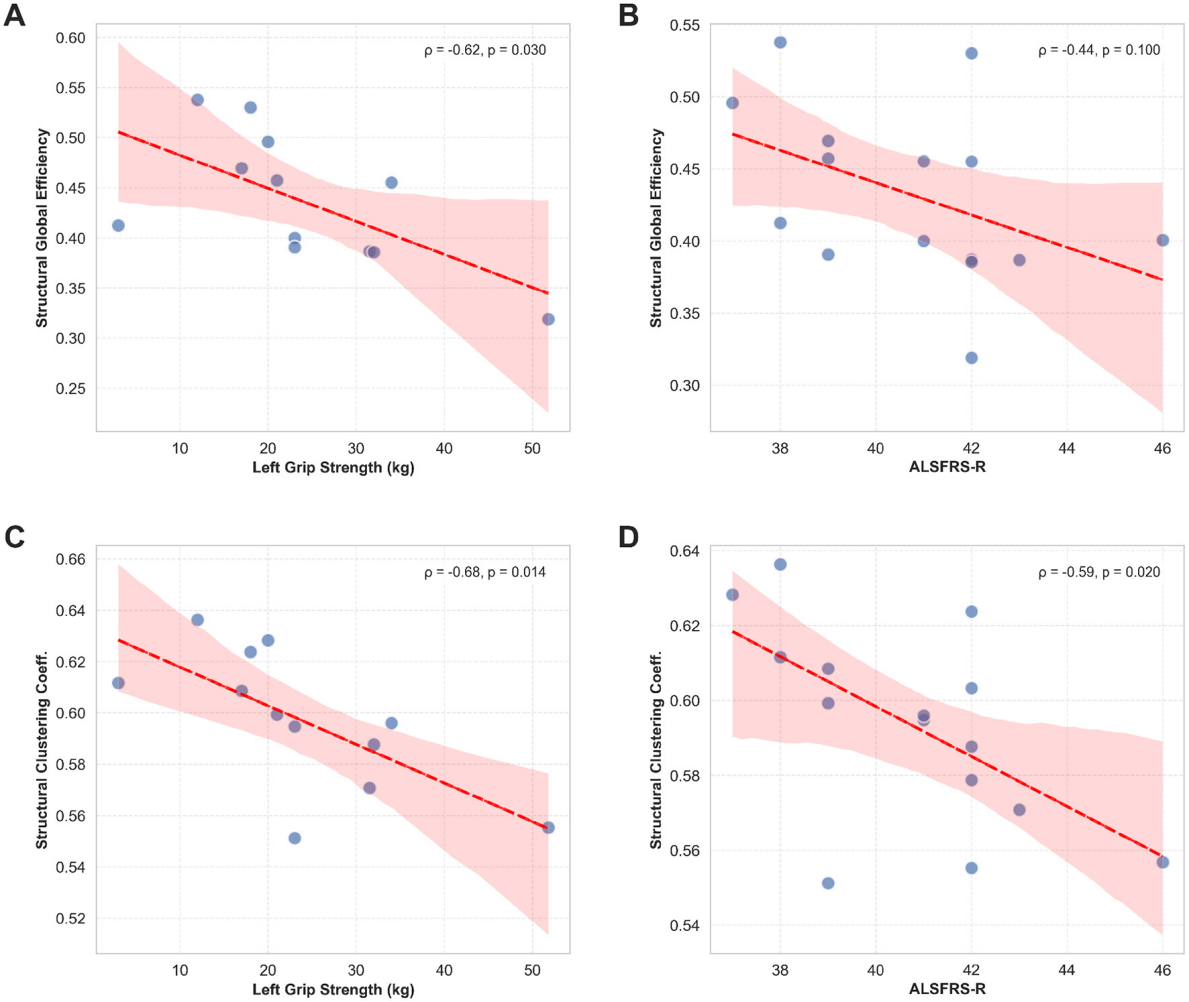
Clinical correlations in ALS patients. Scatterplots showing significant correlations between structural network metrics and clinical measures. **(A)** Structural global efficiency vs. Left Grip Strength (ρ = −0.70, *p* = 0.004). **(B)** Structural global efficiency vs. ALSFRS-R (ρ = −0.56, *p* = 0.031). **(C)** Structural clustering vs. Left Grip Strength (ρ = −0.63, *p* = 0.012). **(D)** Structural clustering vs. ALSFRS-R (ρ = −0.60, *p* = 0.018). Data points represent individual ALS subjects (Blue: *n* = 15). Lines represent linear regression fits. Shaded areas indicate 95% confidence intervals.

These negative correlations suggest that patients with more severe clinical impairment exhibit paradoxically higher structural network efficiency and clustering, possibly reflecting compensatory reorganization or maladaptive plasticity in response to neurodegeneration. In contrast, functional connectivity metrics showed non-significant positive trends with clinical measures (overall connectivity × ALSFRS-R: ρ = 0.47, *p* = 0.078), suggesting different mechanisms underlying structural vs. functional network changes in ALS (Figure 7).

### 3.7 Additional topological network metrics (structural and functional)

To further characterize the topology of the connectome, we calculated additional graph-theoretical metrics including characteristic path length, assortativity coefficient, small-worldness, and rich-club coefficient. Results for both structural (DTI) and functional (Rest and Task fMRI) networks are summarized in Table 6.

No statistically significant group differences were observed for any of these metrics across modalities (all *p* > 0.05). Both ALS and control groups exhibited strong small-world topology (σ » 1) in structural and functional networks, indicating preserved efficient information transfer. Structural networks showed disassortative mixing (hub-to-non-hub connections), whereas functional networks tended toward positive assortativity (hub-to-hub connections). Notably, ALS patients showed a trend toward increased assortativity in resting-state fMRI (*p* = 0.065), possibly reflecting altered hub organization.

### 3.8 Nodal network analysis

To complement global network analyses, we examined node-specific metrics for key motor and frontotemporal regions. We calculated degree, betweenness centrality, and clustering coefficient for 20 anatomically-defined nodes including primary motor cortex (precentral gyrus), primary sensory cortex (postcentral gyrus), premotor regions, and frontotemporal areas (Table 9).

**Table 9:** Nodal metrics for key motor regions (structural and functional).

**Node**	**Metric (modality)**	**ALS**	**Control**	***p*-Value**
L precentral	Degree (DTI)	9.7 ± 3.0	9.7 ± 2.2	0.88
L precentral	Strength (fMRI)	34.96 ± 7.05	31.02 ± 10.55	0.326
R precentral	Degree (DTI)	2.2 ± 2.5	1.9 ± 2.4	0.79
R precentral	Strength (fMRI)	37.68 ± 6.87	32.05 ± 11.03	0.085
L postcentral	Degree (DTI)	6.3 ± 1.8	5.4 ± 1.3	0.13
L postcentral	Strength (fMRI)	28.11 ± 11.02	31.42 ± 9.71	0.183
R postcentral	Degree (DTI)	0.0 ± 0.0	0.0 ± 0.0	1.000
R postcentral	Strength (fMRI)	34.42 ± 9.62	31.85 ± 10.27	0.527

All comparisons non-significant (*p* > 0.05). DTI Degree refers to binary connection count in the top-5% thresholded network; fMRI Strength refers to the sum of absolute correlations (unthresholded) to capture the full dynamic range of functional coupling. Asymmetry in DTI degree (Left > Right) was observed across both groups.

No significant group differences were observed for any nodal metric in any region (all *p* > 0.15). For DTI, the left precentral gyrus showed comparable degree (ALS: 88.0 ± 0.0, Control: 88.0 ± 0.0, *p* = 1.0) and betweenness centrality. Similarly, for fMRI, nodal strength (weighted degree) in the left precentral gyrus was comparable between groups (ALS: 34.96 ± 7.05, Control: 31.02 ± 10.55, *p* = 0.33). Similar patterns were observed across all examined nodes, indicating preserved nodal organization in motor and frontotemporal networks across both structural and functional modalities.

## 4 Discussion

Our findings provide new insights into the network-level reorganization that characterizes ALS. Contrary to the classical view of the disease as a disconnection syndrome marked by global reductions in structure–function coupling, we demonstrate a more nuanced pattern: cortical covariance networks show minimal association with functional connectivity, while white matter–functional coupling remains robust and, in some cases, is even strengthened. This selective reorganization highlights the complex and adaptive nature of the ALS connectome (9, 10).

### 4.1 Selective structure–function decoupling and compensation

The marked dissociation between cortical covariance networks and functional dynamics likely reflects neurodegenerative processes that preferentially affect cortical microstructure and synaptic integrity (11). Cortical thinning, loss of pyramidal neurons, and local disorganization may disrupt the structural substrates underlying long-range functional integration (12). In contrast, the preserved or enhanced coupling between diffusion-based white matter connectivity and functional synchronization suggests that large-scale axonal architecture remains sufficiently intact to support communication between distributed regions, even in the presence of cortical pathology (11, 13).

### 4.2 Comparison with previous studies

Prior investigations into structure–function coupling in ALS have produced mixed results, with some reporting global reductions in correspondence (14), while others note regional variability or preserved connectivity. Our multimodal analysis clarifies these discrepancies by disentangling different structural substrates. Studies focusing solely on cortical thickness or structural covariance may detect profound disorganization, leading to the conclusion of widespread decoupling. However, diffusion-based tractography captures the large-scale white matter architecture that continues to constrain and shape functional communication, explaining the strong correlations observed in our data (15, 16).

Furthermore, the observation that structure–function coupling remains particularly strong during task performance underscores the dynamic nature of this relationship (17). It suggests that disease-related reorganization is not static but context-dependent, with task demands potentially amplifying compensatory connectivity patterns.

### 4.3 Clinical and therapeutic implications

Our analysis revealed an inverse relationship between structural network topology and clinical status, where patients with greater functional impairment (lower ALSFRS-R and grip strength scores) exhibited paradoxically higher global efficiency and clustering coefficients. This finding aligns with the “compensatory hyperconnectivity” hypothesis often described in early-to-mid stage neurodegeneration. As direct structural pathways degenerate, the network may reorganize through the recruitment of alternative, redundant pathways or disinhibition of existing connections to maintain system-wide integration. However, the strong negative correlation with clinical performance suggests that this reorganization may eventually become maladaptive or insufficient to sustain function as the disease burden increases. Alternatively, this could reflect a “rich-get-richer” phenomenon where catastrophic failure of peripheral low-degree nodes leads to an artificial inflation of global efficiency metrics calculated on the remaining, highly connected core network.

The persistence of structure–function coupling—particularly when linked to this hyperconnectivity—has significant clinical implications (18). While compensatory network reorganization may support motor performance in early disease stages, excessive or maladaptive hyperconnectivity could eventually contribute to network instability, excitotoxicity, and disease progression (19). This dual role highlights the potential for therapeutic interventions aimed at modulating network dynamics.

For instance, neuromodulatory strategies such as repetitive transcranial magnetic stimulation (rTMS), transcranial direct current stimulation (tDCS), or targeted pharmacological agents could be employed to rebalance hyperactive networks or reinforce weakened connections (20, 21). Moreover, advanced neurorehabilitation protocols might exploit preserved white matter–functional coupling to enhance plasticity and slow functional decline (22). Cross-modal metrics could also serve as sensitive biomarkers for monitoring disease progression, assessing therapeutic efficacy, and guiding individualized treatment strategies (23).

### 4.4 Limitations and future directions

Our study has several limitations. First, the sample size (*n* = 29), while powered to detect large effects and consistent with similar neuroimaging studies, limits our ability to detect subtle network changes. Second, although we employed a high-resolution 104-node atlas across modalities, probabilistic tractography in cerebellar regions remains challenging due to complex fiber crossing; future studies using high-angular resolution diffusion imaging (HARDI) may allow for even more robust cerebellar structural mapping. Third, this is a cross-sectional study; longitudinal designs are needed to track the trajectory of compensatory reorganization. Finally, while we observed strong structure–function coupling, the exact cellular mechanisms (e.g., specific inhibitory interneuron involvement) remain to be elucidated using animal models or post-mortem pathology.

Finally, while our correlation-based approach captures global structure–function correspondence, it does not fully resolve the complexity of nonlinear or higher-order interactions within the diseased connectome. Emerging computational approaches—including graph neural networks, multivariate machine learning, and deep connectomic modeling—offer exciting opportunities to uncover subtle, spatially distributed patterns of reorganization that may escape conventional analyses. Such techniques, when applied to larger, longitudinal datasets, could further elucidate the progressive remodeling of cortical and subcortical networks in ALS and potentially yield predictive biomarkers for prognosis and therapeutic stratification.

## 5 Conclusion

Taken together, our results refine the conventional model of ALS as a disease of network disintegration. Instead, they suggest a selective and dynamic reorganization of structure–function relationships, with cortical-level decoupling coexisting alongside robust and potentially compensatory white matter–functional coupling. Recognizing and targeting these network-level adaptations could open new avenues for intervention and improve our understanding of disease progression and treatment response.

## Data Availability

The raw data supporting the conclusions of this article will be made available by the authors, without undue reservation.
