# Supplementary material for: Connectivity in ALS II (CoALS II): a study of structural and functional connectivity in ALS
Source: Front Neurol. 2026 Mar 25;17:1743723. doi: 10.3389/fneur.2026.1743723 (PMC13056628; doi:10.3389/fneur.2026.1743723)
Supplement: Supplementary file 1 [file Data_Sheet_1.pdf]

Supplementary Figure S1: Anatomical Reference for ROI Parcellation

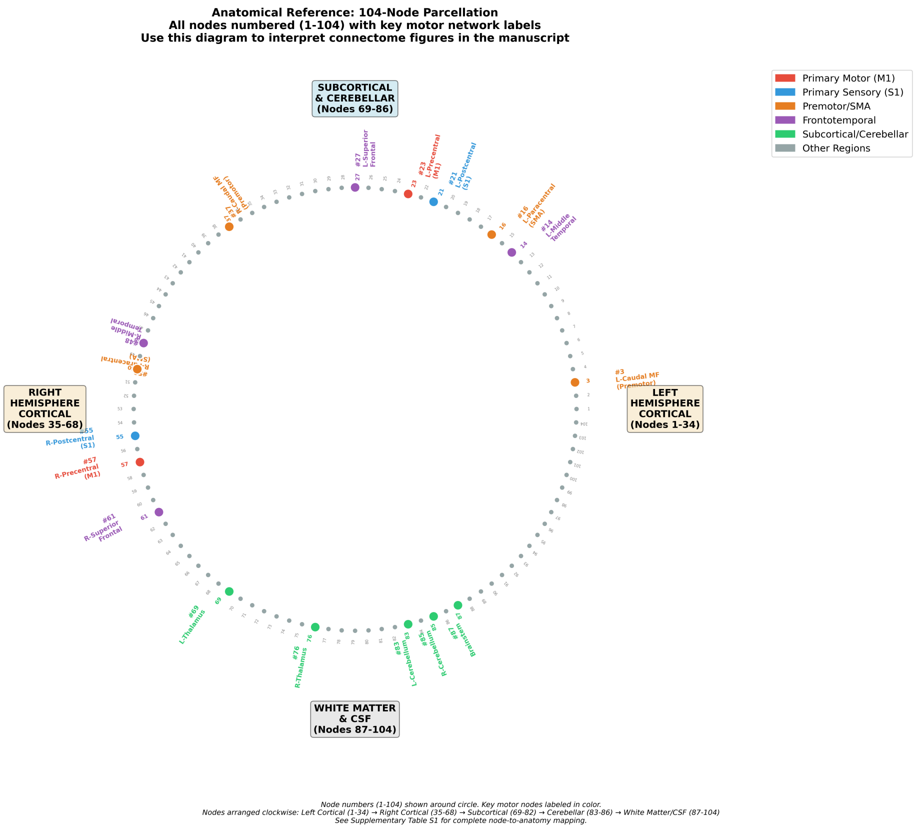

Figure 1: Supplementary Figure S1

Purpose

This reference figure provides anatomical context for interpreting the connectome visualizations (Figures 1-6 in the main manuscript). It maps the 104-node parcellation scheme to anatomical locations, with emphasis on motor and frontotemporal networks relevant to ALS pathophysiology.

Key Motor Network Nodes (Primary Focus for ALS)

Primary Motor Cortex

- **Node 23:** Left Precentral Gyrus (L-PREC) - Left Primary Motor Cortex

- **Node 57:** Right Precentral Gyrus (R-PREC) - Right Primary Motor Cortex

Primary Sensory Cortex

- **Node 21:** Left Postcentral Gyrus (L-POSTC) - Left Primary Somatosensory Cortex
- **Node 55:** Right Postcentral Gyrus (R-POSTC) - Right Primary Somatosensory Cortex

Supplementary Motor Area (SMA) and Premotor

- **Node 16:** Left Paracentral Lobule (L-PARAC) - includes SMA
- **Node 50:** Right Paracentral Lobule (R-PARAC) - includes SMA
- **Node 3:** Left Caudal Middle Frontal (L-CMF) - Premotor Cortex
- **Node 37:** Right Caudal Middle Frontal (R-CMF) - Premotor Cortex
- **Node 26:** Left Rostral Middle Frontal (L-RMF) - Dorsolateral Prefrontal/Premotor
- **Node 60:** Right Rostral Middle Frontal (R-RMF) - Dorsolateral Prefrontal/Premotor

Broca's Area (Language/Motor Speech)

- **Node 17:** Left Pars Opercularis (L-POPER) - Broca's area (part)
- **Node 19:** Left Pars Triangularis (L-PTRI) - Broca's area (part)

Frontotemporal Network Nodes (Relevant for ALS-FTD Spectrum)

Frontal Regions

- **Node 27:** Left Superior Frontal (L-SF)
- **Node 61:** Right Superior Frontal (R-SF)
- **Node 11:** Left Lateral Orbitofrontal (L-LOF)
- **Node 45:** Right Lateral Orbitofrontal (R-LOF)
- **Node 13:** Left Medial Orbitofrontal (L-MOF)
- **Node 47:** Right Medial Orbitofrontal (R-MOF)
- **Node 31:** Left Frontal Pole (L-FP)
- **Node 65:** Right Frontal Pole (R-FP)

Temporal Regions

- **Node 14:** Left Middle Temporal (L-MT)
- **Node 48:** Right Middle Temporal (R-MT)
- **Node 8:** Left Inferior Temporal (L-IT)
- **Node 42:** Right Inferior Temporal (R-IT)

- **Node 29:** Left Superior Temporal (L-ST)
- **Node 63:** Right Superior Temporal (R-ST)
- **Node 32:** Left Temporal Pole (L-TP)
- **Node 66:** Right Temporal Pole (R-TP)

Limbic/Cingulate Regions

- **Node 2:** Left Caudal Anterior Cingulate (L-CAC)
- **Node 36:** Right Caudal Anterior Cingulate (R-CAC)
- **Node 25:** Left Rostral Anterior Cingulate (L-RAC)
- **Node 59:** Right Rostral Anterior Cingulate (R-RAC)
- **Node 22:** Left Posterior Cingulate (L-PC)
- **Node 56:** Right Posterior Cingulate (R-PC)

Subcortical Motor Structures

Basal Ganglia (Motor Control)

- **Node 70:** Left Caudate (L-CAUD)
- **Node 77:** Right Caudate (R-CAUD)
- **Node 71:** Left Putamen (L-PUT)
- **Node 78:** Right Putamen (R-PUT)
- **Node 72:** Left Pallidum (L-PALL)
- **Node 79:** Right Pallidum (R-PALL)

Thalamus (Motor Relay)

- **Node 69:** Left Thalamus (L-THAL)
- **Node 76:** Right Thalamus (R-THAL)

Cerebellum (Motor Coordination)

- **Node 83:** Left Cerebellar Cortex (L-CBLM-CTX)
- **Node 85:** Right Cerebellar Cortex (R-CBLM-CTX)
- **Node 84:** Left Cerebellar White Matter (L-CBLM-WM)
- **Node 86:** Right Cerebellar White Matter (R-CBLM-WM)

Brain Stem (Motor Pathways)

- **Node 87:** Brain Stem (BSTEM) - contains corticospinal tract

---

Parietal Association Areas

Superior Parietal

- **Node 28:** Left Superior Parietal (L-SP)
- **Node 62:** Right Superior Parietal (R-SP)

Inferior Parietal

- **Node 7:** Left Inferior Parietal (L-IP)
- **Node 41:** Right Inferior Parietal (R-IP)

Supramarginal Gyrus

- **Node 30:** Left Supramarginal (L-SMAR)
- **Node 64:** Right Supramarginal (R-SMAR)

Precuneus (Default Mode Network)

- **Node 24:** Left Precuneus (L-PCUN)
- **Node 58:** Right Precuneus (R-PCUN)

Interhemispheric Connections (Corpus Callosum)

White Matter Tracts

- **Node 90:** Corpus Callosum Anterior (CC-ANT)
- **Node 91:** Corpus Callosum Mid-Anterior (CC-MANT)
- **Node 92:** Corpus Callosum Central (CC-CENT)
- **Node 93:** Corpus Callosum Mid-Posterior (CC-MPOST)
- **Node 94:** Corpus Callosum Posterior (CC-POST)

Color Coding Scheme for Connectome Figures

In the main manuscript connectograms (Figures 1-6), connections are color-coded as follows:

By Network Type:

- **Red/Warm colors:** Motor network connections (nodes 3, 16, 21, 23, 26, 37, 50, 55, 57, 60)
- **Orange:** Premotor and supplementary motor connections
- **Blue/Cool colors:** Frontotemporal network connections
- **Green:** Parietal association areas
- **Purple:** Subcortical structures
- **Gray:** Other cortical regions

By Group Comparison:

- **Red ribbons:** Stronger connectivity in ALS group
- **Blue ribbons:** Stronger connectivity in Control group
- **Pink/Purple ribbons:** Overlapping structural-functional connections

---

Spatial Organization in Circular Connectograms

The 104 nodes are arranged clockwise around the circle in the following order:

1. **Left Hemisphere Cortical (Nodes 1-34):** Starting from frontal pole, moving posteriorly through frontal, parietal, occipital, temporal regions
2. **Right Hemisphere Cortical (Nodes 35-68):** Mirror arrangement of left hemisphere
3. **Left Subcortical (Nodes 69-75):** Thalamus, basal ganglia, limbic structures
4. **Right Subcortical (Nodes 76-82):** Mirror of left subcortical
5. **Cerebellar & Brainstem (Nodes 83-87)**
6. **White Matter & CSF (Nodes 88-104)**

Interpreting the Connectograms

Reading Connection Strength:

- **Ribbon thickness:** Proportional to connection strength (streamline count for DTI, correlation coefficient for fMRI)
- **Ribbon color intensity:** Darker = stronger connection
- **Number of ribbons:** More ribbons = more distributed connectivity

Key Patterns to Observe:

In Healthy Controls: - Strong bilateral motor network connectivity (nodes 21, 23, 55, 57) - Robust interhemispheric connections via corpus callosum - Balanced frontotemporal coupling

In ALS Patients: - Selective reduction in motor network connections - Preserved or enhanced cortico-cerebellar pathways (compensation) - Altered interhemispheric motor connectivity - Maintained frontotemporal network structure

In Difference Maps (ALS - Control): - Red ribbons indicate relatively stronger connections in ALS (potential compensation) - Blue ribbons indicate relatively weaker connections in ALS (degeneration) - Focus on motor network (nodes 16, 21, 23, 50, 55, 57) for primary ALS pathology

---

Clinical Relevance of Key Nodes

Upper Motor Neuron Signs:

- **Nodes 23, 57 (Precentral):** Primary motor cortex - weakness, spasticity
- **Nodes 16, 50 (Paracentral/SMA):** Supplementary motor - motor planning deficits

Bulbar Symptoms:

- **Nodes 17, 19 (Broca's area):** Speech and swallowing difficulties
- **Node 87 (Brainstem):** Cranial nerve nuclei involvement

Cognitive/Behavioral (ALS-FTD):

- **Nodes 11, 13, 31, 45, 47, 65 (Orbitofrontal/Frontal Pole):** Executive dysfunction, behavioral changes
- **Nodes 8, 14, 32, 42, 48, 66 (Temporal):** Language, semantic memory

Motor Coordination:

- **Nodes 83, 85 (Cerebellar Cortex):** Compensatory motor control
- **Nodes 69, 76 (Thalamus):** Motor relay and integration

Reference

This parcellation is based on: - **Desikan, R. S., et al. (2006).** An automated labeling system for subdividing the human cerebral cortex on MRI scans into gyral based regions of interest. *NeuroImage*, 31(3), 968-980. - **FreeSurfer anatomical statistics** (https://surfer.nmr.mgh.harvard.edu/)

For complete 104-node mapping, see **Supplementary Table S1**.

Usage Instructions

When viewing connectome figures in the main manuscript:

1. **Identify nodes of interest** using this reference guide
2. **Locate nodes** in the circular arrangement (clockwise from top)
3. **Trace connections** (ribbons) between nodes
4. **Compare groups** (ALS vs. Control) using difference maps
5. **Interpret clinical significance** using the clinical relevance section above

Example: To examine motor network integrity: - Locate nodes 23 and 57 (bilateral precentral gyrus) - Observe connections to nodes 21, 55 (postcentral - sensorimotor integration) - Check connections to nodes 16, 50 (SMA - motor

planning) - Examine interhemispheric connections via corpus callosum (nodes 90-94) - Compare ALS vs. Control to identify degenerative vs. compensatory patterns
