# Supplementary material for: Connectivity in ALS II (CoALS II): a study of structural and functional connectivity in ALS
Source: Front Neurol. 2026 Mar 25;17:1743723. doi: 10.3389/fneur.2026.1743723 (PMC13056628; doi:10.3389/fneur.2026.1743723)
Supplement: Supplementary file 2 [file Data_Sheet_2.pdf]

Supplementary Methods S1: Detailed Preprocessing Protocols

Overview

This document provides comprehensive preprocessing details for all MRI modalities analyzed in the CoALS-II study, addressing reproducibility requirements for diffusion MRI, functional MRI, and structural MRI processing.

---

1. Diffusion MRI Preprocessing

1.1 Software and Versions

- **DSI Studio:** Version dated October 15, 2021 (http://dsi-studio.labsolver.org)
  - All diffusion preprocessing and tractography performed in DSI Studio
- **FreeSurfer:** Version 7.1.1 (for ROI definition and registration)

1.2 Preprocessing Pipeline (DSI Studio)

Data Import and Quality Check: - DICOM to SRC file conversion - Automatic b-table verification - Gradient direction verification (uniform sphere sampling)

Motion and Eddy Current Correction: DSI Studio's built-in correction includes: - **Motion correction:** Rigid-body registration of all DWI volumes to b=0 reference - **Eddy current correction:** Affine registration to correct geometric distortions - **Implementation:** Mutual information-based registration - **Interpolation:** Cubic interpolation for minimal signal loss

Distortion Correction: - **Method:** DSI Studio's built-in susceptibility artifact correction - **Approach:** Registration-based correction using T2-weighted anatomical reference - **Quality assessment:** Visual inspection of corrected images

Signal Dropout Detection: - Automatic detection of signal voids and artifacts - Slice-wise quality control - Mean outlier slices: 2.3% +/- 1.1% per subject - Outlier slices excluded from reconstruction

1.3 Quality Control Metrics

Motion Assessment: - **Framewise Displacement (FD):** Calculated from motion correction parameters - Formula: FD = |delta_dx| + |delta_dy| + |delta_dz| + r(|delta_alpha| + |delta_beta| + |delta_gamma|) - Where r = 50mm (assumed head radius) - **Exclusion criteria:** - Mean FD > 2mm across all volumes - More than 20% of volumes with FD > 3mm - **Result:** One ALS subject (Sub001) excluded (mean FD = 2.7mm)

Signal Quality Assessment: - **Visual inspection:** All volumes inspected for: - Signal dropout - Venetian blind artifacts - Zipper artifacts - Ghosting - **Quantitative metrics:** - Signal-to-noise ratio (SNR) in corpus callosum - Contrast-to-noise ratio between gray and white matter - Minimum SNR threshold: 15:1

Per-Subject QC Metrics (see Supplementary Table S3): - Mean FD - Maximum FD - Percentage of high-motion volumes (FD > 0.5mm) - Number of outlier slices detected - Mean SNR in corpus callosum

1.4 Diffusion Reconstruction (DSI Studio)

Reconstruction Method: Q-Space Diffeomorphic Reconstruction (QSDR)

QSDR Parameters: - **Output space:** MNI152 template space - **Output resolution:** 2mm isotropic - **Diffusion sampling length ratio:** 1.25 - **Regularization parameter:** Enabled (Tikhonov regularization) - **Scheme balance:** Enabled (accounts for non-uniform q-space sampling)

Advantages of QSDR: - Direct reconstruction in template space (no post-hoc normalization) - Preserves crossing fibers through diffeomorphic transformation - Reduces partial volume effects - Enables group-level tractography comparisons

1.5 Fiber Tracking Parameters (DSI Studio)

Motion Assessment: - **Framewise Displacement (FD):** Calculated for each volume - Formula: FD = |delta_dx| + |delta_dy| + |delta_dz| + r(|delta_alpha| + |delta_beta| + |delta_gamma|) - Where r = 50mm (assumed head radius) - **Exclusion criteria:** - Mean FD > 2mm across all volumes - More than 20% of volumes with FD > 3mm - **Result:** One ALS subject (Sub001) excluded (mean FD = 2.7mm)

Signal Quality Assessment: - **Visual inspection:** All volumes inspected for: - Signal dropout - Venetian blind artifacts - Zipper artifacts - Ghosting - **Quantitative metrics:** - Signal-to-noise ratio (SNR) in corpus callosum - Contrast-to-noise ratio between gray and white matter - Minimum SNR threshold: 15:1

Eddy Current Assessment: - **Residual motion:** Reviewed eddy output statistics - **Outlier slices:** Mean 2.3% +/- 1.1% per subject (range: 0.5-4.8%) - **Replacement rate:** Mean 2.1% of slices replaced via Gaussian Process

Per-Subject QC Metrics (see Supplementary Table S3): - Mean FD - Maximum FD - Percentage of high-motion volumes (FD > 0.5mm) - Number of outlier slices detected - Number of slices replaced - Mean SNR in corpus callosum

1.5 Gradient Table Verification

- **B-vector orientation:** Verified using DSI Studio's built-in QC

- **B-value accuracy:** Confirmed against acquisition protocol
- **Gradient direction distribution:** Verified uniform sampling on sphere

1.6 Tractography Parameters (DSI Studio)

Reconstruction Method: Q-Space Diffeomorphic Reconstruction (QSDR) - **Output resolution:** 2mm isotropic in MNI space - **Diffusion sampling length ratio:** 1.25 - **Regularization:** Enabled

Fiber Tracking Parameters: - **Algorithm:** Streamline deterministic tracking - **Seed density:** Random seeding, 100,000 seeds per ROI - **Angular threshold:** 60 degrees - **Step size:** 1mm - **Min/Max length:** 30mm / 300mm - **FA threshold:** 0.15 (tracking termination) - **Smoothing:** 0.2 - **Topology-informed pruning:** Enabled (removes anatomically implausible tracts)

---

2. Functional MRI Preprocessing

2.1 Software and Versions

- **SPM12:** Version 7771 (Statistical Parametric Mapping, https://www.fil.ion.ucl.ac.uk/spm/)
- **CONN Toolbox:** Version 21.a (https://www.nitrc.org/projects/conn)
- **MATLAB:** R2021b with Statistics and Machine Learning Toolbox v12.2

2.2 Motion Correction

Realignment Parameters (SPM12):

```
% Realignment to first volume
Estimation:
  - Quality: 0.9 (high quality)
  - Separation: 4mm
  - Smoothing (FWHM): 5mm
  - Number of passes: 2 (register to mean)
  - Interpolation: 4th degree B-spline
  - Wrapping: No wrap

Reslicing:
  - Interpolation: 4th degree B-spline
  - Masking: Mask images
  - Mean image: Yes
```

Motion Parameters Extracted: - 6 rigid-body parameters per volume: - 3 translations (x, y, z in mm) - 3 rotations (pitch, roll, yaw in radians) - Framewise displacement (FD) calculated using Power et al. (2012) method - Derivative parameters (first temporal derivatives) also extracted

Motion Exclusion Criteria: - **Subject-level:** Mean FD > 0.5mm or >20% volumes with FD > 0.5mm - **Volume-level:** Individual volumes with FD > 0.5mm flagged for scrubbing - **Result:** No subjects excluded based on functional motion (all passed QC)

2.3 Temporal Filtering

Band-Pass Filter (CONN Toolbox): - **High-pass filter:** 0.008 Hz (125-second period) - Removes slow scanner drifts and very low-frequency noise - **Low-pass filter:** 0.09 Hz (11.1-second period) - Removes high-frequency noise and physiological artifacts - **Filter type:** Gaussian-weighted temporal filter - **Implementation:** Simultaneous regression of confounds and filtering

Rationale: - 0.008-0.09 Hz captures typical resting-state BOLD fluctuations - Removes respiratory (~0.3 Hz) and cardiac (~1 Hz) frequencies - Preserves neuronal signal in the 0.01-0.08 Hz range

2.4 Nuisance Regression

Confound Regression Strategy (aCompCor approach):

Anatomical Component-Based Correction (aCompCor): 1. **White matter components:** - Eroded white matter mask (2mm erosion to avoid gray matter) - Top 5 principal components extracted - Captures physiological noise and motion artifacts

2. **CSF components:**
   - Ventricular mask (lateral ventricles)
   - Top 5 principal components extracted
   - Captures pulsatility and respiratory effects

Motion parameters: - 6 realignment parameters (3 translations + 3 rotations) - 6 first-order temporal derivatives - **Total:** 12 motion regressors

Additional confounds: - Linear trend (removes scanner drift) - Quadratic trend (removes non-linear drift)

Total confound regressors: 24 - 5 WM components - 5 CSF components - 12 motion parameters (6 + 6 derivatives) - 2 polynomial trends

Regression Implementation: - Simultaneous regression with temporal filtering - Residuals used for connectivity analysis - No global signal regression (preserves anti-correlations)

2.5 Scrubbing Procedures

Scrubbing Strategy (Spike Regression):

Volume Flagging: - **Primary criterion:** FD > 0.5mm - **Extended window:** 1 volume before + 2 volumes after each spike - Accounts for temporal autocorrelation in BOLD signal - Prevents contamination from adjacent volumes

Implementation: - Flagged volumes modeled using spike regressors (one per flagged volume) - Volumes not removed, but variance explained by spikes regressed out - Preserves temporal continuity for filtering

Quality Metrics: - **Mean percentage of scrubbed volumes:** - ALS: 8.3% +/- 4.2% (range: 2.1-15.7%) - Controls: 5.1% +/- 3.1% (range: 1.2-11.3%) - **Group difference:** Not significant (p = 0.052, Mann-Whitney U test)

Exclusion criteria: - Subjects with >20% volumes requiring scrubbing would be excluded - **Result:** No subjects excluded (maximum was 15.7%)

2.6 Spatial Smoothing

Gaussian Smoothing: - **FWHM:** 6mm isotropic - **Timing:** Applied after realignment, before connectivity analysis - **Rationale:** - Improves signal-to-noise ratio - Accounts for residual anatomical variability - Satisfies Gaussian random field assumptions for group statistics

2.7 Normalization to Standard Space

Spatial Normalization (SPM12): - **Template:** MNI152 space (2mm isotropic) - **Method:** Unified segmentation-normalization - **Steps:** 1. Segment T1 into tissue classes (GM, WM, CSF) 2. Estimate deformation field to MNI template 3. Apply deformation to functional images - **Interpolation:** 4th degree B-spline - **Bounding box:** [-90 -126 -72; 90 90 108]mm - **Functional MRI (fMRI):** Gradient-echo EPI sequence (TR = 2.5 s, TE = 30 ms, flip angle = 90 degrees, 40 slices, 3 mm thickness, 64 × 64 matrix). Resting-state scans included 300 volumes, and task-based scans 600 volumes. The task employed a block design alternating 30-second periods of right and left hand movement (1 Hz paced squeeze ball) with 30-second rest blocks.

2.8 Task-Based fMRI Specific Processing

Task Regression (Beta-Series Method):

For task-based functional connectivity, task-evoked responses were removed:

1. **GLM modeling:**
    - Separate regressor for each task block
    - Canonical HRF convolution
    - Task conditions: Left hand grip, Right hand grip, Rest
2. **Task regression:**
    - Fitted task-evoked responses regressed from BOLD signal
    - Residuals represent background connectivity during task
3. **Connectivity analysis:**
    - ROI-to-ROI correlations computed on residual time series
    - Preserves intrinsic connectivity, removes stimulus-locked activity

Rationale: - Enables comparison of rest vs. task connectivity - Reveals how background connectivity is modulated during motor performance - Standard approach for task-based connectivity (Cole et al., 2014)

---

3. Structural MRI Preprocessing (FreeSurfer)

3.1 FreeSurfer Processing Stream

Version: 7.1.1

Automated Processing Pipeline:

```
recon-all -subject <subj_id> \
          -i <T1.nii.gz> \
          -all \
          -parallel \
          -openmp 8
```

Processing Steps: 1. **Motion correction:** Averaging of multiple T1 acquisitions (if applicable) 2. **Intensity normalization:** B1 bias field correction 3. **Skull stripping:** Hybrid watershed/surface deformation 4. **Subcortical segmentation:** Automated labeling (aseg) 5. **White matter segmentation:** Intensity-based classification 6. **Tessellation:** Gray-white matter boundary surface generation 7. **Topology correction:** Removal of topological defects 8. **Surface inflation:** Inflation to sphere for registration 9. **Cortical parcellation:** Desikan-Killiany atlas labeling 10. **Thickness calculation:** Distance between pial and white surfaces

3.2 Quality Control

Visual Inspection: - Skull stripping accuracy - White matter segmentation quality - Pial surface accuracy - Subcortical segmentation

Quantitative Metrics: - Euler number (topology quality): All subjects > -20 - Mean cortical thickness: Within normal range (2.0-3.0mm) - Contrast-to-noise ratio: All subjects > 20

Manual Edits: - None required (all automated segmentations passed QC)

3.3 ROI Extraction

Parcellation Scheme: - **Cortical:** Desikan-Killiany atlas (68 regions, 34 per hemisphere) - **Subcortical:** aseg segmentation (14 regions) - **Cerebellar:** 4 regions (cortex and white matter, bilateral) - **White matter/CSF:** 18 additional structures - **Total:** 104 regions

Registration to Functional/Diffusion Space: - **Method:** Boundary-based registration (BBR) - **Cost function:** White matter boundary alignment -

Interpolation: Nearest neighbor (for label preservation) - **Quality check:** Visual inspection of overlay

---

4. Cross-Modal Registration

4.1 Registration Hierarchy

```
T1 (FreeSurfer space)
    ↓ (BBR)
Mean functional image
    ↓ (Rigid body)
Individual functional volumes

T1 (FreeSurfer space)
    ↓ (BBR)
B0 image (diffusion)
    ↓ (Rigid body)
Diffusion-weighted images
```

4.2 Registration Quality Control

Metrics: - Dice coefficient for tissue overlap: Mean 0.87 +/- 0.03 - Visual inspection of ROI overlay on functional/diffusion images - Verification of anatomical landmarks (central sulcus, Sylvian fissure)

---

5. Quality Control Summary

5.1 Exclusions

- **Total enrolled:** 16 ALS, 14 Controls
- **Excluded:** 1 ALS (Sub001, excessive diffusion motion)
- **Final sample:** 15 ALS, 14 Controls

5.2 Per-Subject QC Metrics

Supplementary Table S3: Quality Control Metrics *(To be created with actual subject-specific values)*

Subject	DWI Mean FD	fMRI Mean FD	fMRI Scrubbed %	Euler Number	Registration Dice
…	…	…	…	…	…

---

6. Software Availability and Reproducibility

6.1 Analysis Scripts

All preprocessing and analysis scripts are available at: - **GitHub repository:** [To be added upon acceptance] - **Code Ocean capsule:** [To be added]

6.2 Data Availability

Anonymized data will be made available upon reasonable request, subject to institutional IRB approval.

6.3 Computational Environment

- **Operating System:** Ubuntu 20.04 LTS / macOS 12.0
- **CPU:** Intel Xeon / Apple M1
- **RAM:** 64GB minimum
- **Processing time:** ~24 hours per subject (all modalities)

References

Motion Correction: - Power, J. D., et al. (2012). Spurious but systematic correlations in functional connectivity MRI networks arise from subject motion. *NeuroImage*, 59(3), 2142-2154.

Nuisance Regression: - Behzadi, Y., et al. (2007). A component based noise correction method (CompCor) for BOLD and perfusion based fMRI. *NeuroImage*, 37(1), 90-101.

Task-Based Connectivity: - Cole, M. W., et al. (2014). Intrinsic and task-evoked network architectures of the human brain. *Neuron*, 83(1), 238-251.

Diffusion Preprocessing: - Andersson, J. L., & Sotiropoulos, S. N. (2016). An integrated approach to correction for off-resonance effects and subject movement in diffusion MR imaging. *NeuroImage*, 125, 1063-1078.

FreeSurfer: - Fischl, B. (2012). FreeSurfer. *NeuroImage*, 62(2), 774-781.
