# Supplementary material for: Connectivity in ALS II (CoALS II): a study of structural and functional connectivity in ALS
Source: Front Neurol. 2026 Mar 25;17:1743723. doi: 10.3389/fneur.2026.1743723 (PMC13056628; doi:10.3389/fneur.2026.1743723)
Supplement: Supplementary file 3 [file Data_Sheet_3.pdf]

Supplementary Table S1: Complete ROI Mapping for 104-Node Parcellation

Desikan-Killiany Atlas with Subcortical and Cerebellar Regions

Node	Anatomical Region	Hemisphere	Lobe/Region	Abbreviation
1	Banks of Superior Temporal Sulcus	Left	Temporal	L-BSTS
2	Caudal Anterior Cingulate	Left	Frontal	L-CAC
3	Caudal Middle Frontal	Left	Frontal	L-CMF
4	Cuneus	Left	Occipital	L-CUN
5	Entorhinal	Left	Temporal	L-ENT
6	Fusiform	Left	Temporal	L-FUS
7	Inferior Parietal	Left	Parietal	L-IP
8	Inferior Temporal	Left	Temporal	L-IT
9	Isthmus Cingulate	Left	Limbic	L-ISTC
10	Lateral Occipital	Left	Occipital	L-LO
11	Lateral Orbitofrontal	Left	Frontal	L-LOF
12	Lingual	Left	Occipital	L-LING
13	Medial Orbitofrontal	Left	Frontal	L-MOF
14	Middle Temporal	Left	Temporal	L-MT
15	Parahippocampal	Left	Temporal	L-PARAH
16	Paracentral	Left	Frontal	L-PARAC
17	Pars Opercularis	Left	Frontal	L-POPER
18	Pars Orbitalis	Left	Frontal	L-PORB
19	Pars Triangularis	Left	Frontal	L-PTRI
20	Pericalcarine	Left	Occipital	L-PERIC
21	Postcentral	Left	Parietal	L-POSTC
22	Posterior Cingulate	Left	Limbic	L-PC
23	Precentral	Left	Frontal	L-PREC
24	Precuneus	Left	Parietal	L-PCUN
25	Rostral Anterior Cingulate	Left	Frontal	L-RAC
26	Rostral Middle Frontal	Left	Frontal	L-RMF
27	Superior Frontal	Left	Frontal	L-SF
28	Superior Parietal	Left	Parietal	L-SP
29	Superior Temporal	Left	Temporal	L-ST
30	Supramarginal	Left	Parietal	L-SMAR

Node	Anatomical Region	Hemisphere	Lobe/Region	Abbreviation
31	Frontal Pole	Left	Frontal	L-FP
32	Temporal Pole	Left	Temporal	L-TP
33	Transverse Temporal	Left	Temporal	L-TT
34	Insula	Left	Insular	L-INS
35	Banks of Superior Temporal Sulcus	Right	Temporal	R-BSTS
36	Caudal Anterior Cingulate	Right	Frontal	R-CAC
37	Caudal Middle Frontal	Right	Frontal	R-CMF
38	Cuneus	Right	Occipital	R-CUN
39	Entorhinal	Right	Temporal	R-ENT
40	Fusiform	Right	Temporal	R-FUS
41	Inferior Parietal	Right	Parietal	R-IP
42	Inferior Temporal	Right	Temporal	R-IT
43	Isthmus Cingulate	Right	Limbic	R-ISTC
44	Lateral Occipital	Right	Occipital	R-LO
45	Lateral Orbitofrontal	Right	Frontal	R-LOF
46	Lingual	Right	Occipital	R-LING
47	Medial Orbitofrontal	Right	Frontal	R-MOF
48	Middle Temporal	Right	Temporal	R-MT
49	Parahippocampal	Right	Temporal	R-PARAH
50	Paracentral	Right	Frontal	R-PARAC
51	Pars Opercularis	Right	Frontal	R-POPER
52	Pars Orbitalis	Right	Frontal	R-PORB
53	Pars Triangularis	Right	Frontal	R-PTRI
54	Pericalcarine	Right	Occipital	R-PERIC
55	Postcentral	Right	Parietal	R-POSTC
56	Posterior Cingulate	Right	Limbic	R-PC
57	Precentral	Right	Frontal	R-PREC
58	Precuneus	Right	Parietal	R-PCUN
59	Rostral Anterior Cingulate	Right	Frontal	R-RAC
60	Rostral Middle Frontal	Right	Frontal	R-RMF
61	Superior Frontal	Right	Frontal	R-SF
62	Superior Parietal	Right	Parietal	R-SP
63	Superior Temporal	Right	Temporal	R-ST
64	Supramarginal	Right	Parietal	R-SMAR
65	Frontal Pole	Right	Frontal	R-FP
66	Temporal Pole	Right	Temporal	R-TP

Node	Anatomical Region	Hemisphere	Lobe/Region	Abbreviation
67	Transverse Temporal	Right	Temporal	R-TT
68	Insula	Right	Insular	R-INS
69	Thalamus	Left	Subcortical	L-THAL
70	Caudate	Left	Subcortical	L-CAUD
71	Putamen	Left	Subcortical	L-PUT
72	Pallidum	Left	Subcortical	L-PALL
73	Hippocampus	Left	Subcortical	L-HIPP
74	Amygdala	Left	Subcortical	L-AMYG
75	Accumbens	Left	Subcortical	L-ACCU
76	Thalamus	Right	Subcortical	R-THAL
77	Caudate	Right	Subcortical	R-CAUD
78	Putamen	Right	Subcortical	R-PUT
79	Pallidum	Right	Subcortical	R-PALL
80	Hippocampus	Right	Subcortical	R-HIPP
81	Amygdala	Right	Subcortical	R-AMYG
82	Accumbens	Right	Subcortical	R-ACCU
83	Cerebellar Cortex	Left	Cerebellar	L-CBLM-CTX
84	Cerebellar White Matter	Left	Cerebellar	L-CBLM-WM
85	Cerebellar Cortex	Right	Cerebellar	R-CBLM-CTX
86	Cerebellar White Matter	Right	Cerebellar	R-CBLM-WM
87	Brain Stem	Bilateral	Brain Stem	BSTEM
88	Ventral Diencephalon	Left	Subcortical	L-VDIEN
89	Ventral Diencephalon	Right	Subcortical	R-VDIEN
90	Corpus Callosum Anterior	Bilateral	White Matter	CC-ANT
91	Corpus Callosum Mid-Anterior	Bilateral	White Matter	CC-MANT
92	Corpus Callosum Central	Bilateral	White Matter	CC-CENT
93	Corpus Callosum Mid-Posterior	Bilateral	White Matter	CC-MPOST
94	Corpus Callosum Posterior	Bilateral	White Matter	CC-POST
95	Cerebral White Matter	Left	White Matter	L-CWM
96	Cerebral White Matter	Right	White Matter	R-CWM
97	Lateral Ventricle	Left	CSF	L-LATVENT

Node	Anatomical Region	Hemisphere	Lobe/Region	Abbreviation
98	Lateral Ventricle	Right	CSF	R-LATVENT
99	Third Ventricle	Bilateral	CSF	3RD-VENT
100	Fourth Ventricle	Bilateral	CSF	4TH-VENT
101	CSF	Bilateral	CSF	CSF
102	Choroid Plexus	Left	CSF	L-CHPL
103	Choroid Plexus	Right	CSF	R-CHPL
104	Optic Chiasm	Bilateral	White Matter	OPTIC-CHSM

Notes

Cortical Regions (Nodes 1-68): Based on the Desikan-Killiany atlas, which parcellates the cortical surface into 34 regions per hemisphere (68 total).

Subcortical Structures (Nodes 69-82): Deep gray matter structures including thalamus, basal ganglia, hippocampus, and amygdala.

Cerebellar Regions (Nodes 83-86): Cerebellar cortex and white matter for both hemispheres.

Brain Stem (Node 87): Midbrain, pons, and medulla.

White Matter and CSF Structures (Nodes 88-104): Additional structures including corpus callosum segments, cerebral white matter, ventricles, and other CSF spaces.

Key Motor Network Nodes (Relevant for ALS)

- **Node 23 (L-PREC):** Left Precentral Gyrus (Primary Motor Cortex)
- **Node 57 (R-PREC):** Right Precentral Gyrus (Primary Motor Cortex)
- **Node 21 (L-POSTC):** Left Postcentral Gyrus (Primary Sensory Cortex)
- **Node 55 (R-POSTC):** Right Postcentral Gyrus (Primary Sensory Cortex)
- **Node 16 (L-PARAC):** Left Paracentral Lobule (includes Supplementary Motor Area)
- **Node 50 (R-PARAC):** Right Paracentral Lobule (includes Supplementary Motor Area)
- **Node 3 (L-CMF):** Left Caudal Middle Frontal (Premotor Cortex)
- **Node 37 (R-CMF):** Right Caudal Middle Frontal (Premotor Cortex)

Key Frontotemporal Network Nodes (Relevant for ALS-FTD)

- **Node 27 (L-SF):** Left Superior Frontal
- **Node 61 (R-SF):** Right Superior Frontal

- **Node 26 (L-RMF):** Left Rostral Middle Frontal
- **Node 60 (R-RMF):** Right Rostral Middle Frontal
- **Node 14 (L-MT):** Left Middle Temporal
- **Node 48 (R-MT):** Right Middle Temporal
- **Node 8 (L-IT):** Left Inferior Temporal
- **Node 42 (R-IT):** Right Inferior Temporal

Reference

This parcellation scheme is based on: - Desikan, R. S., et al. (2006). An automated labeling system for subdividing the human cerebral cortex on MRI scans into gyral based regions of interest. *NeuroImage*, 31(3), 968-980. - FreeSurfer anatomical statistics and segmentation protocols (https://surfer.nmr.mgh.harvard.edu/)
