# Supplementary figures and images for: Connectivity in ALS II (CoALS II): a study of structural and functional connectivity in ALS
Source: Front Neurol. 2026 Mar 25;17:1743723. doi: 10.3389/fneur.2026.1743723 (PMC13056628; doi:10.3389/fneur.2026.1743723)

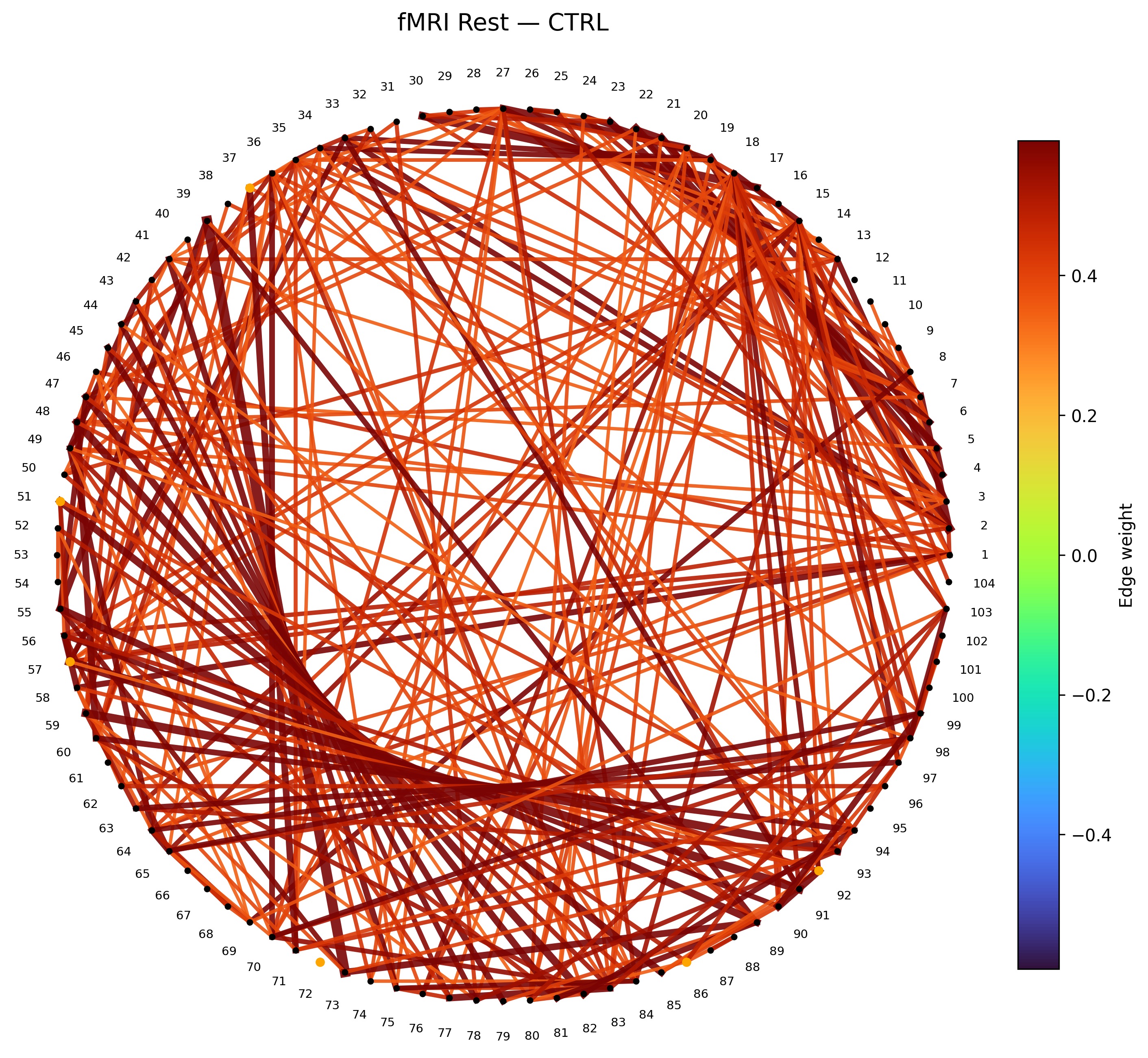

Supplement: Supplementary file 5 [file Image_1.jpeg]
